# Effect of Tempering Temperature on the Aqueous Corrosion Resistance of 9Cr Series Heat-Resistant Steel

**DOI:** 10.3390/ma17204960

**Published:** 2024-10-11

**Authors:** Hui Li, Hao Bai

**Affiliations:** 1 School of Metallurgical and Ecological Engineering, University of Science and Technology Beijing, 30 Xueyuan Road, Beijing 100083, China; 2 China Metallurgical Industry Planning and Research Institute, 36 North 3rd Ring East Road, Beijing 100013, China

**Keywords:** aqueous corrosion resistance, heat-resistant steel, tempering temperature, precipitate

## Abstract

In this investigation, the aqueous corrosion resistance of 9Cr series heat-resistant steel during tempering was investigated. Optical Microscopy (OM), Scanning Electron Microscopy (SEM), and Energy Dispersive Spectrometer (EDS) were used to analyze the effect of tempering temperature on the microstructure and precipitation behavior of precipitates. The heat-resisting steel was heated to 1150 °C for 1 h, and then tempered at different temperatures between 680 °C and 760 °C for 2 h. The microstructure of the heat-resistant steel after tempering was composed of lath-tempered martensite and fine precipitates. The hardness decreased with increasing tempering temperature, ranging from HBW 261 to HBW 193. The aqueous corrosion resistance improved as the tempering temperatures increased from 680 °C to 720 °C but deteriorated at higher temperatures, such as 760 °C, which was obtained by an electrochemical corrosion performance test. The aqueous corrosion resistance was affected by the decrease in dislocation density and the decrease in Cr solution in the tempered martensite. With the increase in the tempering temperature, the aqueous corrosion potential first increases and then decreases, the self-corrosion current density first decreases and then increases, and the polarization resistance first increases and then decreases. Furthermore, the increase in corrosion resistance is attributed to the reduction in dislocation density and chromium depletion in the martensitic structure as the tempering temperature approaches 720 °C. This paper reveals the effect of tempering temperature on the corrosion resistance of 9Cr series heat-resistant steel, which is a further exploration of a known phenomenon.

## 1. Introduction

The new 9Cr series ferritic heat-resistant steel was developed by strengthening the alloying of T122 steel and T9 steel [1,2]. The high steam operation parameters of thermal power units require that heat-resistant steel components not only have good creep resistance but also have good aqueous corrosion resistance [3,4]. Therefore, the research on the aqueous corrosion resistance of heat-resistant steel was very important in the development of materials for thermal power units [5,6,7].

Intergranular corrosion is a common local corrosion phenomenon of metal materials, which is not easy to find. Avoiding intergranular corrosion is the key intention of corrosion-resistant materials research and development [8,9]. The high-temperature creep property of heat-resistant steels is usually improved by increasing the content of the Cr element. The high-Cr heat-resistant steel precipitates a large amount of M_23_C_6_ (a carbide, M is Cr and a small amount of Fe) during tempering and forms a Cr-poor zone around it [4,10,11]. The poor Cr zone in heat-resistant steel is corroded easily and forms intergranular corrosion.

It was found that [12] the aqueous corrosion cracking of the intergranular stress caused by the intergranular aqueous corrosion behavior of the pipe eventually leads to the failure of the pipe. Shot peening on the surface of the pipe can induce the martensite to precipitate a large number of fine carbides and reduce the influence of the Cr-poor zone. The optimization of the forming composition and the improvement of the forming process can avoid Cr-poor zones and the intergranular corrosion of metal materials [13]. The study of aqueous corrosion products was an important reference for showing the corrosion mechanism of heat-resistant steel. The reason for corrosion failure can be analyzed by studying the morphology, composition, and formation process of corrosion products [14,15].

It was found [16,17] that the pore formation mechanism of P92 steel after oxidative corrosion was not caused by the volatilization of corrosion products but by the loose structure of corrosion products. The corrosion mechanism of TP347H heat-resistant steel was due to the excessive coefficient of the thermal expansion of the heat-resistant steel itself, leading to the cracking and detachment of the surface oxide layer [18,19]. The Cr content in the heat-resistant steel was high, so the surface easily formed dense Cr_2_O_3_ to achieve the effect of corrosion resistance, which was similar to the corrosion resistance principle of stainless steel [20,21]. When a large number of precipitates containing Cr are precipitated from heat-resistant steel, the Cr_2_O_3_ oxide layer with a dense surface will be seriously damaged, which also becomes the main reason for the corrosion failure of heat-resistant steel [6,22]. Based on the above research, it can be seen that the Cr element in heat-resistant steel is the main factor affecting corrosion resistance. The existing form of Cr element in steel is mainly affected by tempering heat treatment, so the choice of heat treatment temperature plays an important role in improving corrosion resistance.

The influence of Al content in heat-resistant steel on aqueous corrosion resistance cannot be ignored. It was found that X10CrAlSi18 steel with high Al content has a significantly lower oxidation weight gain and oxidation rate than low Al heat-resistant steel during the high-temperature oxidation process [23]. The addition of 0.3% Si content in HTUPS4 steel [24] promotes the precipitation of B2-NiAl, destroys the surface Al_2_O_3_ oxide layer, and significantly reduces the high-temperature oxidation resistance [25,26].

The fine precipitates in heat-resistant steel are one of the main means to improve the creep resistance at high temperatures. The precipitation of Cr-containing carbides at the grain boundaries and lath boundaries (a martensitic structure characterized by lath morphology) of heat-resistant steel during tempering is an important means to stabilize the tempering of martensitic structures at high temperatures [27,28,29]. The precipitation of Cr-containing carbides in heat-resistant steel has an important effect on the aqueous corrosion resistance [30]. In summary, this study investigates the effect of tempering temperature on the precipitation of Cr-containing carbides and its impact on the corrosion resistance of 9Cr series steel. The novelty of this study lies in exploring a particular range of tempering temperatures, and a new understanding of Cr precipitation and corrosion resistance.

## 2. Experimental Procedure

### 2.1. Material Preparation

The 9Cr series ferritic heat-resistant ingot (Fe-0.12C-0.2Si-1.0Mn-9.67Cr-2.0Mo-1.32Co-0.18Ni-0.3V-0.04Nb-0.01N-0.005B, all in mass %) was obtained by using a vacuum induction furnace. The material was heated at 1150 °C for 1 h, followed by air cooling [31]. Afterwards, it was tempered at different temperatures ranging from 680 °C to 760 °C for 2 h [32].

### 2.2. Microstructure Analysis

Optical Microscopy (OM) (OLYMPUS, Tokyo, Japan), Scanning Electron Microscopy (SEM) (JSM-IT800, Japan Electronics Co., Ltd., Tokyo, Japan), and Energy Dispersive Spectrometer (EDS) were used to analyze the effect of tempering temperature on the microstructure and precipitation behavior of precipitates. The microstructure characterizations were carried out using OM, SEM [33], and EDS. The number and size of the precipitates in the SEM image were counted using the Image-Pro Plus 6.0 (IPP) software.

### 2.3. Mechanical Testing

The surface hardness of the tempered heat-resistant steel samples was tested using a Brinell hardness (Future-Tech, Kawasaki, Japan) tester with a 10 mm diameter ball indenter and a 3000 kg load, and five measurements were averaged for each sample.

### 2.4. Corrosion Testing

All of the samples were placed into a temperature and humidity chamber (Beijing Yashilin Test Equipment Co., Ltd., Beijing, China) for 30 days under a temperature and relative humidity of 45 °C and 95%, respectively. After 10, 20, and 30 days, the samples were removed from the chamber and observed using SEM to analyze surface corrosion topographies. The average aqueous corrosion rates of the samples were evaluated based on their potentiodynamic polarization in a 3.5 wt% NaCl solution using an electrochemical workstation (Shanghai zero Dew instrument equipment Co., Ltd., Shanghai, China) at ambient temperature. A conventional three-electrode system—with a platinum plate as the counter electrode, a saturated calomel electrode as the reference electrode, and a surface-untreated heat-resisting steel sample as the working electrode—was used. Prior to potentiodynamic polarization measurement, the open circuit potential was monitored for approximately 30 min. The applied potential was swept from −0.2 V to 0.3 V at a scan rate of 0.5 mV/s, and the polarization was terminated when the self-corrosion current density reached 10^−2^ A/cm^2^. Finally, the applied potential versus the logarithm of the corresponding self-corrosion current density was plotted to obtain a polarization curve [5]. The potentiodynamic polarization measurements were conducted on three replicate samples for each tempering. Untreated heat-resistant steel samples were tested alongside the tempered samples to provide a baseline for comparison.

## 3. Results and Discussion

### 3.1. Microstructure Observation

#### 3.1.1. Microstructure Observation before Tempering

**Figure 1:**
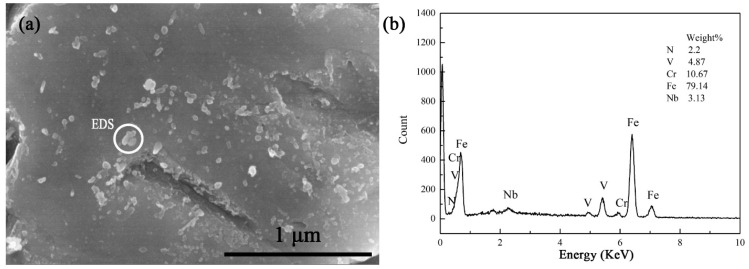
SEM image of heat-resistant steel before tempering. (**a**) SEM; (**b**) EDS.

The microstructure of heat-resistant steel before tempering was lath-tempered martensite and fine precipitate, as shown in Figure 1a.

BN inclusions were not observed in the SEM images of the heat-resistant steel before tempering, while some nanosized Nb and V precipitates were observed in Figure 1b. The dissolution of BN inclusion in heat-resistant steel is a prerequisite for Nb and V precipitation. The dissolution of BN inclusion provides an opportunity for the formation of B-containing carbides during tempering.

#### 3.1.2. Microstructure after Tempering

**Figure 2:**
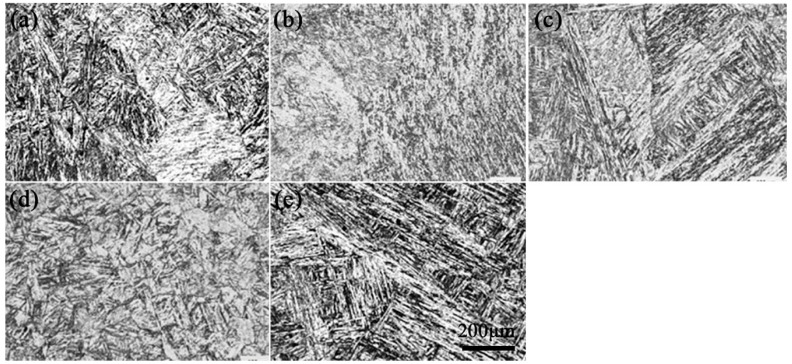
OM image after different tempering temperatures of heat-resistant steel: (**a**) 680 °C; (**b**) 700 °C; (**c**) 720 °C; (**d**) 740 °C; (**e**) 760 °C.

The OM image of the heat-resistant steel after tempering at 680 °C to 760 °C is shown in Figure 2.

The structure of the heat-resistant steel was tempered martensite lath (TML) after tempering. There were several martensite blocks with different orientations in a primary austenite grain. In the process of melting and solidification, the heat-resistant steel was prone to forming defects such as inclusion, segregation, and shrinkage. Various oxidizing inclusions were important factors affecting the mechanical properties and aqueous corrosion resistance of the heat-resistant steel.

**Figure 3:**
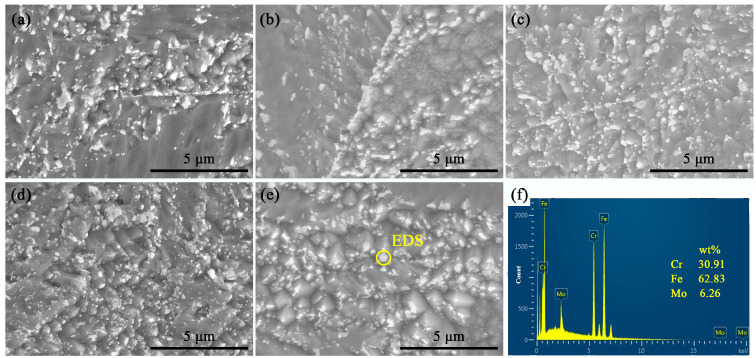
SEM image of heat-resistant steel after tempering at different temperatures (**a**) 680 °C; (**b**) 700 °C; (**c**) 720 °C; (**d**) 740 °C; (**e**) 760 °C; (**f**) EDS.

The SEM image of the heat-resistant steel after tempering at 680 °C to 760 °C is shown in Figure 3.

The primary austenite grain boundaries and precipitates were clearly observed in the heat-resistant steels after different tempering. The size of the precipitates on the grain boundary was obviously larger than that inside the grain. The grain boundary position provides more powerful growth conditions for the precipitates.

### 3.2. Hardness

**Figure 4:**
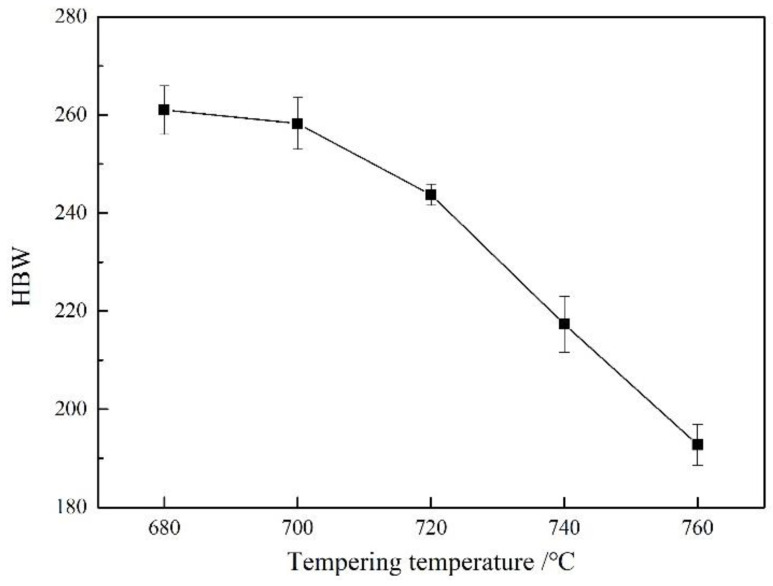
Hardness of heat-resistant steel after different tempering.

The influence of the tempering temperature on the hardness of the heat-resistant steel is shown in Figure 4.

With the tempering temperature increasing from 680 °C to 760 °C, the average hardness of the heat-resistant steel was HBW 261, HBW 258, HBW 243, HBW 217, and HBW 193, respectively. With the increase in the tempering temperature, the hardness of the heat-resistant steel decreased as a whole. The increase in the tempering temperature reduced the dislocation density and quenching hardness in the matrix, which was the main reason for the decrease in precision of the heat-resistant steel.

### 3.3. Electrochemical Aqueous Corrosion Performance

**Figure 5:**
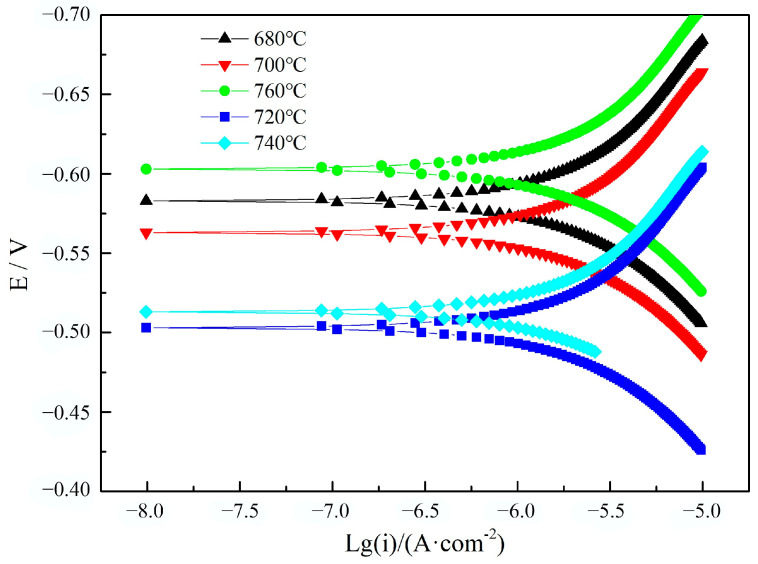
Polarization curve diagram of heat-resistant steel after different tempering.

The potentiodynamic polarization curve of the tempered heat-resistant steel in the 3.5 wt.%NaCl solution is shown in Figure 5.

The aqueous corrosion potential of the heat-resistant steels with tempering temperatures of 680 °C and 760 °C was the lowest. Tempering at 680 °C and 760 °C gave the heat-resistant steels the worst aqueous corrosion resistance. The self-corrosion current density of the heat-resistant steels after different tempering in the 3.5 wt.%NaCl solution is shown in Table 1.

The self-corrosion current density of the heat-resistant steels tempered at 680 °C and 760 °C was higher than other samples. The heat-resistant steels tempered at 720 °C and 740 °C had a lower self-corrosion current density than the other samples.

**Figure 6:**
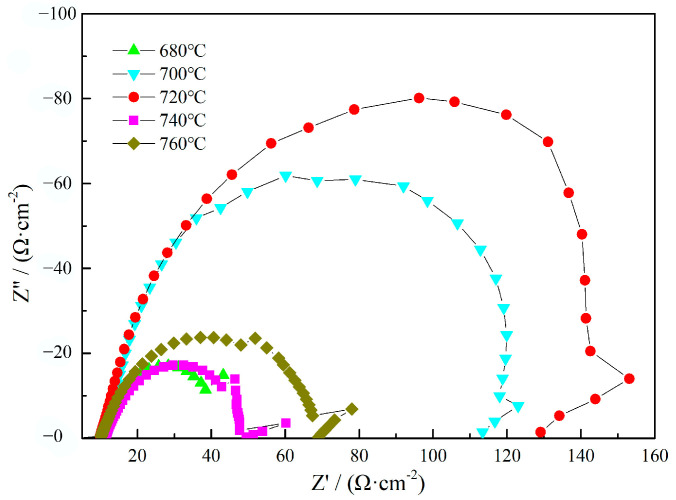
Impedance diagram of heat-resistant steel after treatment with different tempering.

The electrochemical impedance diagram of the heat-resistant steel after tempering is shown in Figure 6.

The shape of the impedance spectra of the heat-resistant steels with different tempering temperatures was similar. The impedance maps were of a single semi-arc shape. Charge movement was the main factor for the aqueous corrosion behavior of the heat-resistant steel in the electrochemical aqueous corrosion process. When the arc radius of the impedance spectrum was larger, it indicated that the charge transfer speed was smaller, so the protection performance of the passivation film formed on the surface of the heat-resistant steel was better. The arc radius of the impedance spectrum of the heat-resistant steel increased first and then decreased when the tempering temperature increased from 680 °C to 760 °C. The corrosion resistance of the heat-resistant steel increased first and then decreased when the tempering temperature increased from 680 °C to 760 °C. When the tempering temperature was 720 °C, the arc radius of the impedance spectrum of the heat-resistant steel was the largest, indicating that the heat-resistant steel tempered at 720 °C has the best corrosion resistance.

With the help of the Zsimpewin software (Version 3.3), the impedance map of the heat-resistant steel in the 3.5 wt.%NaCl solution was fitted with an equivalent circuit diagram, and the fitting results are shown in Table 2. The polarization resistance value of the heat-resistant steel increased first and then decreased with the increase in the tempering temperature. The polarization resistance of the heat-resistant steel was 138.87 Ω·cm^2^. The aqueous corrosion resistance of the heat-resistant steel was optimal when the tempering temperature was 720 °C.

### 3.4. Aqueous Corrosion Behavior in Neutral Salt Spray Environment

**Figure 7:**
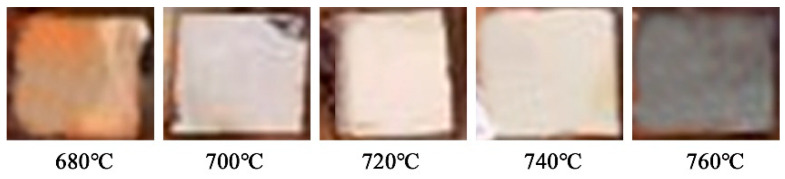
Macro image of heat-resistant steel samples after 30 days.

The macromorphology of the tempered heat-resistant steel after 30 days in a neutral salt spray environment is shown in Figure 7.

The heat-resistant steel sample was surrounded by resin except for the polishing surface. The polished surface of heat-resistant steel tempered at 680 °C and 760 °C had the most obvious aqueous corrosion. The polished surface of the heat-resistant steel tempered at 700 °C to 740 °C was not obviously corroded. This experimental result was consistent with the results of previous electrochemical aqueous corrosion tests. The aqueous corrosion of heat-resistant steel in a neutral salt spray environment starts with pitting. The pitting position of the specimen surface was convex, which was the trace of aqueous corrosion. The corroded areas of the heat-resistant steel were covered with reddish-brown, dark red, and black aqueous corrosion products. The inclined placement of the heat-resistant steel sample caused the lower end to be corroded more significantly.

**Figure 8:**
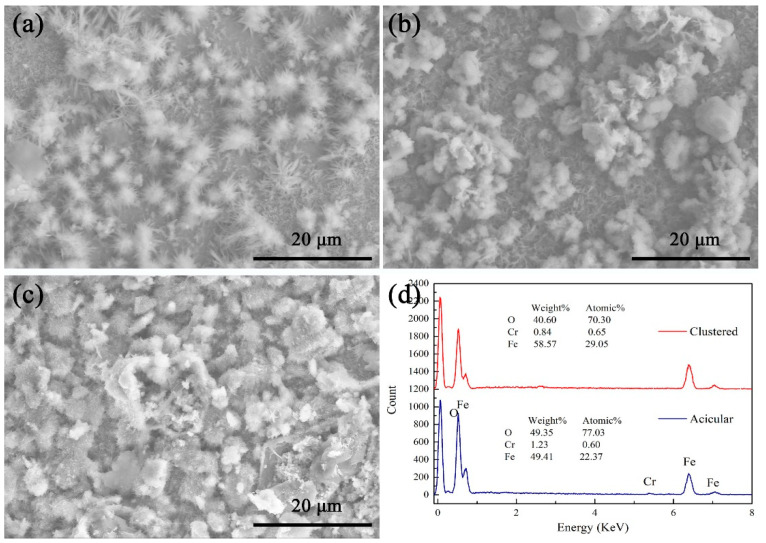
SEM image of heat-resistant steel surface after neutral salt spray aqueous corrosion: (**a**) day 10; (**b**) day 20; (**c**) day 30; (**d**) EDS.

The SEM images of the corrosion products of the heat-resistant steel tempered at 720 °C in a neutral salt spray environment for 10 days, 20 days, and 30 days, respectively, are shown in Figure 8.

A large number of acicular aqueous corrosion products were present on the surface after another ten days (Figure 8a). At the initial stage of corrosion, the corrosion products on the surface increased with time. Acicular corrosion products were loosely deposited on the surface of the heat-resistant steel. The acicular corrosion products were caused by the aqueous corrosion of Cl^−^, O^2^, and water vapor on the surface of the heat-resistant steel.

After 20 days of aqueous corrosion, a large number of acicular aqueous corrosion products were gradually replaced by clustered aqueous corrosion products (Figure 8b). The aqueous corrosion products on the surface changed from loose to dense. After 20 days of aqueous corrosion, the acicular aqueous corrosion products did not completely transform into cluster-like corrosion products. After 30 days of corrosion, the surface of the heat-resistant steel was covered by a cluster of corrosion products. The surface of the heat-resistant steel was surrounded by a dense, clustered corrosion layer.

### 3.5. Discussion

**Figure 9:**
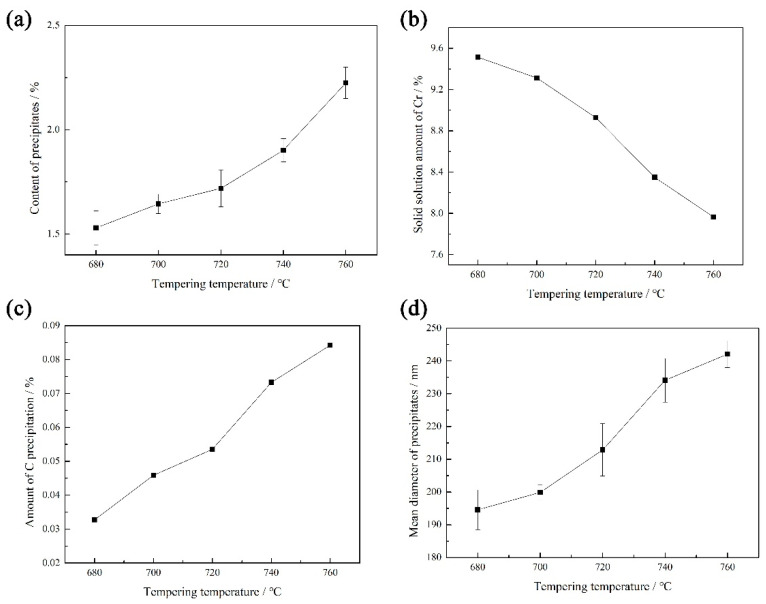
Effect of the tempering temperature on the precipitate and element content in the heat-resistant steel: (**a**) the amount of precipitates; (**b**) the solid solution amount of Cr; (**c**) the amount of C precipitation; (**d**) the average size of the precipitates.

The effect of the tempering temperature on the average size of the precipitate and the solid solution amount of the Cr element in the heat-resistant steel is shown in Figure 9.

*Influence law*

With the increase in the tempering temperature, the average size and amount of the precipitate increased obviously. When the tempering temperature increased from 680 °C to 760 °C, the average size of the precipitate in the heat-resistant steel was 195 nm, 200 nm, 213 nm, 234 nm, and 242 nm, respectively. The precipitation amounts were 1.53%, 1.64%, 1.72%, 1.90%, and 2.22%, respectively, as the tempering temperature increased.

*Cr elements and carbides*

With the tempering temperature increasing from 680 °C to 760 °C, the solid solution of the Cr element in the heat-resistant steel was 9.51%, 9.31%, 8.93%, 8.35%, and 7.67%, while the precipitation of the C element was 0.327‰, 0.459‰, 0.535‰, 0.733‰, and 0.842‰, respectively. The hardness of the tempered heat-resistant steel was inversely proportional to the precipitation of the C element. The hardness of the heat-resistant steel after quenching was proportional to the solid solution of the C element. When the precipitation of the C element in the heat-resistant steel increased after tempering, the hardness value of the heat-resistant steel decreased.

*Dislocation density*

The high-density dislocation region is a high-energy region prone to dissolution during the corrosion process and promotes the development of corrosion [34,35]. The dislocation density of the heat-resistant steel after quenching was proportional to the solid solution content of the C element. The aqueous corrosion resistance of the heat-resistant steel was inversely proportional to the dislocation density. Therefore, with the increase in the tempering temperature, the aqueous corrosion resistance of the heat-resistant steel matrix increased with the decrease in the solid solution of the C element. On the other hand, with the increase in the tempering temperature, the solid solution of the Cr element in the heat-resistant steel decreased. The aqueous corrosion resistance of the heat-resistant steel was proportional to the solid solution content of the Cr element in the matrix. Cr in a solid solution contributes to the formation of a protective Cr_2_O_3_ oxide layer, which enhances corrosion resistance. However, excessive precipitation of Cr-containing carbides can reduce the amount of Cr available for oxide formation, lowering the corrosion resistance. Therefore, the aqueous corrosion resistance of the heat-resistant steel decreased with the increase in the tempering temperature. With the increase in the tempering temperature, the aqueous corrosion resistance of the heat-resistant steel first increased and then decreased due to the influence of the solid solution content of the C element and Cr element in the matrix.

## 4. Conclusions

In this investigation, the effect of the tempering temperature on the aqueous corrosion resistance of 9Cr series heat-resistant steel and the major conclusions obtained are as follows:

1. The microstructure of the heat-resistant steel after tempering was composed of lath-tempered martensite and fine precipitates. With the tempering temperature increasing from 680 °C to 760 °C, the average size and amount of the precipitates increased slowly at first and then significantly.
2. The aqueous corrosion resistance of the heat-resistant steel decreased with the decrease in the Cr content in the solid solution and increased with the decrease in the dislocation density. With the increase in the tempering temperature, the solid solution Cr content and the dislocation density continued to decrease, which is the main reason for the increase and then decrease in corrosion resistance. The aqueous corrosion resistance of the heat-resistant steel tempered at 720 °C was the best. The tempering temperature of 720 °C provides a certain reference value for the industrial production of 9Cr heat-resistant steel.
3. The transformation of surface oxide morphology from acicular to cluster correlates with improved stability and reduced susceptibility to localized corrosion at 720 °C.

## Figures and Tables

**Table 1:** Potentiodynamic polarization curve fitting data of heat-resistant steel in 3.5 wt.%NaCl solution.

Tempering Temperature/°C	680	700	720	740	760
Corrosion Current Density/(μA·cm^−2^)	0.692	0.653	0.612	0.619	0.704

**Table 2:** Fitting results of EIS spectra of heat-resistant steel in 3.5 wt.%NaCl solution.

Tempering Temperature/ °C	Solution Resistance Rs/(Ω·cm^2^)	Charge Transfer Resistance Rt/(Ω·cm^2^)	Film Resistance Rf/(Ω·cm^2^)	Polarization Resistance Rp/(Ω·cm^2^)
680	8.68	22.87	73.51	107.61
700	8.74	15.55	87.82	113.84
720	9.73	31.89	124.9	138.87
740	9.88	19.04	75.83	115.82
760	9.95	15.43	89.43	114.27

## Data Availability

The original contributions presented in the study are included in the article, further inquiries can be directed to the corresponding author.
